# The Major Capsid Protein, VP1, of the Mouse Polyomavirus Stimulates the Activity of Tubulin Acetyltransferase 1 by Microtubule Stabilization

**DOI:** 10.3390/v12020227

**Published:** 2020-02-18

**Authors:** Lenka Horníková, Kateřina Bruštíková, Boris Ryabchenko, Ilia Zhernov, Martin Fraiberk, Zuzana Mariničová, Zdeněk Lánský, Jitka Forstová

**Affiliations:** 1 Department of Genetics and Microbiology, Faculty of Science, Charles University, BIOCEV, 25250 Vestec, Czech Republic; horniko1@natur.cuni.cz (L.H.); katerina.podolska@natur.cuni.cz (K.B.); boris.ryabchenko@natur.cuni.cz (B.R.); martin.fraiberk@natur.cuni.cz (M.F.); zuzana.marinicova@tu-dresden.de (Z.M.); 2 Institute of Biotechnology of the Czech Academy of Sciences, BIOCEV, 25250 Vestec, Czech Republic; Ilia.Zhernov@ibt.cas.cz (I.Z.); zdenek.lansky@ibt.cas.cz (Z.L.); 3 Faculty of Mathematics and Physics, Charles University, 12844 Prague, Czech Republic

**Keywords:** mouse polyomavirus, VP1, microtubules, α-tubulin acetyltransferase 1, histone deacetylase 6, microtubule acetylation, microtubule stabilization

## Abstract

Viruses have evolved mechanisms to manipulate microtubules (MTs) for the efficient realization of their replication programs. Studying the mechanisms of replication of mouse polyomavirus (MPyV), we observed previously that in the late phase of infection, a considerable amount of the main structural protein, VP1, remains in the cytoplasm associated with hyperacetylated microtubules. VP1–microtubule interactions resulted in blocking the cell cycle in the G2/M phase. We are interested in the mechanism leading to microtubule hyperacetylation and stabilization and the roles of tubulin acetyltransferase 1 (αTAT1) and deacetylase histone deacetylase 6 (HDAC6) and VP1 in this mechanism. Therefore, HDAC6 inhibition assays, αTAT1 knock out cell infections, in situ cell fractionation, and confocal and TIRF microscopy were used. The experiments revealed that the direct interaction of isolated microtubules and VP1 results in MT stabilization and a restriction of their dynamics. VP1 leads to an increase in polymerized tubulin in cells, thus favoring αTAT1 activity. The acetylation status of MTs did not affect MPyV infection. However, the stabilization of MTs by VP1 in the late phase of infection may compensate for the previously described cytoskeleton destabilization by MPyV early gene products and is important for the observed inhibition of the G2→M transition of infected cells to prolong the S phase.

## 1. Introduction

The mouse polyomavirus (MPyV) belongs to the *Polyomaviridae* family, a group of non-enveloped, tumorigenic viruses. The virus’s genome is arranged in one molecule of circular dsDNA associated with histones (except histone H1) and encodes six gene products, three early antigens (large, middle and small T) and three structural proteins (the major capsid protein, VP1, and the minor capsid proteins, VP2 and VP3). VP2 is a longer variant of VP3 with a unique prolonged N-terminus. Early antigens are essential for productive virus replication. They participate in viral transcription and DNA replication and deregulate infected cells to ensure a suitable environment for the progression of a virus’s replicative cycle.

The protein capsid with icosahedral symmetry is composed of 72 capsomeres. Each capsomere is built of molecules of VP1 arranged into pentamers, and each pentamer is associated with one molecule of either the VP2 or VP3 minor protein [1]. The minor proteins are not exposed on the surface of the capsid shell. Capsomeres are formed immediately after their synthesis in the cytoplasm, and the complex is transported into the nucleus, where the assembly of virions takes place. Although each structural protein possesses its own nuclear localization signal (NLS), individual expression leads to predominantly cytoplasmic localization. For the successful transport of structural proteins into the nucleus, the capsomere conformation and cooperation of the NLS of the major and the minor structural proteins are absolutely essential [2,3]. The VP1 protein has the ability to self-assemble into capsid-like structures known as virus-like particles (VLPs). Neither minor proteins nor VP1 posttranslational modifications of the VP1 protein are needed for the formation of VLPs [4].

A great deal is known about the interaction of VP1 with the MPyV ganglioside receptor during the virus’s entry and genome delivery to the cell nucleus [5]. However, interactions of VP1 with cellular structures and their significance to infection are not well characterized. Several cellular proteins have been described to interact with VP1 during infection. VP1 interacts with importins, which mediate not only the nuclear transport of capsid proteins during virion assembly but also ensure the translocation of the viral genome from the cytoplasm to the nucleus during the early stages of virus infection [2,6]. In the nucleus, VP1 interacts with the multifunctional cellular transcription factor YY1 [7] and poly(ADP-ribose) polymerase 1 (PARP-1) [8]. It has been suggested that these proteins may be involved in viral transcription and replication regulation and also in viral uncoating and morphogenesis [8,9]. The cellular chaperone, heat shock cognate protein 70 (Hsc70), interacts with VP1 in the cytoplasm immediately after VP1 synthesis and translocates with capsomeres into the nucleus. It was proposed that VP1-Hsc70 interaction prevents the formation of empty capsids in the cytoplasm [10]. The presence of VP1 in cells also affects the posttranslational modification of α-tubulin, namely its acetylation with lysine 40 (αK40) [3]. This modification is localized in the hollow lumen of the microtubules [11] and ensures the resistance of the microtubules to mechanical damage [12,13,14]. Thus, αK40 is a marker of stable microtubules. The primary enzyme responsible for αK40 acetylation is α-tubulin acetyltransferase 1 (αTAT1) [15,16,17]. This enzyme preferentially acetylates polymerized microtubules over tubulin dimers [17]. Deacetylation is maintained by histone deacetylase 6 (HDAC6) [18], which prefers tubulin dimers as a substrate [19].

In our previous study [3], we identified microtubules (including the mitotic spindle) and the cellular chaperone heat shock protein 90 (Hsp90) as other VP1-interacting partners. During the late phase of infection, VP1 can be seen in the cytoplasm decorating microtubules. Complexes of VP1 and microtubules are highly insoluble during in vitro fractionation. We showed that dynein was not responsible for the VP1–microtubule association, and, although Hsp90 could be found in VP1-microtubule complexes, it did not mediate the interaction between VP1 and the microtubules. Nevertheless, the mechanism of VP1–microtubule formation remains obscure. We also observed microtubule hyperacetylation in MPyV infected cells and in cells producing VP1 only.

MPyV gene products affect the cell cycle. Cells infected by MPyV pass through at least two cell cycles [20], but they exhibit a prolonged S phase and no G2→M transition [21]. Dahl et al. [21] showed that cell cycle arrest is connected with the replication of viral DNA, which activates the ATM (protein kinase ataxia telangiectasia mutated) pathway, leading to the inactivation of CDK1 and thus induces cell cycle arrest. We found that the interaction of VP1 with microtubule structures in the cytoplasm resulted in a cell cycle block in the G2/M phase [3]. While early antigens are apparently involved in the first cell cycle block, VP1 may contribute to blocking the second cell cycle, thus enabling the completion of the virus assembly.

In this study, we focus on understanding the relationship of VP1–microtubule binding, microtubule stabilization, and tubulin αK40 acetylation. In addition, we study the relevance of αK40 acetylation for MPyV infection. We revealed that, despite increasing levels of microtubule acetylation in the late phase of MPyV infection, the levels of HDAC6 and αTAT1 expression were not affected. We further showed, during in vitro experiments, that VP1 interacts with microtubules directly and increases their stability. The stabilization of microtubules by VP1 was also proven in VP1-producing cells. Based on our results, we propose that VP1 binding to microtubules leads to microtubule stabilization, which increases the amount of αTAT1 substrate and stimulates αTAT1 activity. Thus, the hyperacetylation of microtubules seems to be a consequence of their stabilization mediated by VP1. However, the microtubule acetylation status per se had no significant effect on MPyV infection.

## 2. Materials and Methods

### 2.1. Cells and Viruses

3T6 (ATCC; CCL-96) mouse fibroblasts and WOP cells [22] (mouse fibroblasts that constitutively produce large T antigens of the mouse polyomavirus) were grown at 37 °C in a 5% CO_2_-air humidified incubator using Dulbecco’s modified Eagle’s medium (DMEM; Sigma-Aldrich, Saint Louis, MO, USA) supplemented with 10% bovine serum (Thermo Fisher Scientific, Waltham, MA, USA) and 2 mM glutamine (Thermo Fisher Scientific). TAT KO cells [23], mouse fibroblasts with αTAT knock out, and their wild-type counterparts, 3T3-wt cells, were grown at 37 °C in a 5% CO_2_-air humidified incubator using DMEM (Sigma-Aldrich) supplemented with 10% bovine serum (Thermo Fisher Scientific), 2 mM glutamine (Thermo Fisher Scientific), 1% non-essential amino acids (Sigma-Aldrich), and 5 mM β-mercaptoethanol (Sigma-Aldrich). SF9 (*Spodoptera frugiperda)* insect cells (ATCC; CRL-1711) were cultivated as adherent cultures at 27 °C in a TNM-FH medium (Sigma-Aldrich) containing 10% bovine serum (Thermo Fisher Scientific) supplemented with 4 mM l-glutamine (Thermo Fisher Scientific). For the stable expression of αTAT1 fused with enhanced green fluorescent protein (EGFP), WOP cells were transfected with pEGFP-αTAT. The WOP-EGFP-αTAT1 cell line was established by sub-cloning and maintained upon blasticidin (InvivoGen, San Diego, CA, USA) selection antibiotics in a DMEM culture medium supplemented with 10% bovine serum (Thermo Fisher Scientific) and 2 mM glutamine (Thermo Fisher Scientific). Mouse polyomavirus (BG strain) was isolated and purified from infected 3T6 cells using the standard protocol [24]. Recombinant baculovirus AcDB3/VP1/EGFP-t-VP3 [25] was propagated using the standard protocol. For infection, the cells were incubated for 1 h (h) with a virus inoculum under a multiplicity of infection (MOI) with 10 plaque forming units per cell.

### 2.2. Plasmids

For the expression of VP1, the plasmids pwP (a gift from Christopher Buck; Addgene plasmid #22519, Addgene, Cambridge, MA, USA) [26] and pVP1 [27] were used. As a control vector, the plasmid pCont [3] was used. The plasmid pEGFP-αTAT1 was constructed using the LR recombination of the donor plasmid pENTR- EGFP-αTAT1 and the destination vector pEF-DEST51 (Thermo Fisher Scientific) according to the manufacturer’s protocol. The donor vector was constructed as follows. The sequence of αTAT1, fused at its N-terminus with EGFP, was amplified by PCR from plasmid pEF5B-FRT-GFP-αTAT1 (a gift from Maxence Nachury; Addgene plasmid #27099) [17] using primer set 5′-CACCATGGTGAGCAAGGGCGAGGAG-3′ and 5′-TTAGTATCGACTCTCCTCAGAG-3′ and inserted into the donor vector pENTR/D-TOPO (Thermo Fisher Scientific) according to the manufacturer’s protocol.

### 2.3. Antibodies

The primary antibodies used were the mouse monoclonal antibody for VP1 [28], rabbit polyclonal antibody for VP1 (prepared in our laboratory), rat monoclonal antibody for LT [29], mouse monoclonal antibody for α-tubulin (Exbio, Prague, Czech Republic or Sigma-Aldrich), mouse monoclonal antibody for acetylated α-tubulin (Sigma-Aldrich), mouse monoclonal antibody for β-tubulin (Sigma-Aldrich), goat polyclonal antibody for biotin (Sigma-Aldrich), rabbit polyclonal antibody for glyceraldehyde 3-phosphate dehydrogenase (GAPDH; Sigma-Aldrich) and rabbit polyclonal antibody for GFP (Abcam, Cambridge, UK). The secondary antibodies used were goat anti-mouse and goat anti-rabbit antibodies conjugated with peroxidase (both from Bio-Rad, Hercules, CA, USA), the donkey anti-rat antibody conjugated with peroxidase (Santa Cruz Biotechnology, Dallas, TX, USA), the donkey anti-rabbit antibody conjugated with Alexa Fluor-488, the goat anti-mouse antibody conjugated with Cy3, the goat anti-rat antibody conjugated with Alexa Fluor-488 (all from Thermo Fisher Scientific), the goat anti-rabbit antibody conjugated with 5-nm gold particles and the goat anti-mouse antibody conjugated with 5-nm gold particles (both BBI Solutions, Cardiff, UK).

### 2.4. Transfection of Cells

The transfection of WOP or WOP-EGFP-αTAT1 cells was performed by electroporation in a Nucleofector™ device using Nucleofector V solution (Lonza, Basel, Switzerland) according to the manufacturer’s instructions. Briefly, 4 × 10^6^ exponentially growing cells were mixed with 6 µg of plasmid DNA and 100 µL of Nucleofector V solution and electroporated (program U-030).

### 2.5. Quantitative Real-Time Polymerase Chain Reaction (qPCR)

The total RNA from 5 × 10^5^ cells was isolated using the High Pure RNA Isolation Kit (Roche) according to the manufacturer’s protocol. Reverse transcription was carried out with the iScriptcDNA Synthesis Kit (Bio-Rad Laboratories) according to the producer’s manual. cDNA was amplified by a polymerase chain reaction using the primer sets HDAC6 (transcription variant 1 a 2), 5′-CGGCTGGTAGATGCACTCAT-3′ and 5′-TGAGAACCCTCTGAATGCGG-3′; αTAT1 (transcription variant 1 and 2), 5′-TTTGAGGATGCAAAGCAACCG-3′ and 5′AGTCCAGAATGCAAAGGGGTT-3′; GAPDH, 5′-ATGACATCAAGAAGGTGGTG-3′ and 5′-CATACCAGGAAATGAGCTTG-3′. The quantification of the PCR products in real time was performed in a Light Cycler 480 II from Roche using the Light Cycler 480 SYBR Green I Master Kit, according to the manufacturer’s protocol. The quantification of target gene expression was performed using Light Cycler 480 II software based on the relative quantification method, thus determining the concentration of target amplicons normalized to the reference GAPDH gene. The fold change of RNA was compared with that of the mock-infected cells.

### 2.6. In Situ Fractionation

In situ fractionation was performed according to [30]. Cells grown on 3 cm Petri dishes were washed three times with a KM buffer (10 mM MES, pH = 6.2; 10 mM NaCl; 1.5 mM MgCl_2_; 10% glycerol; a cocktail of protease inhibitors (Roche)). For the first extraction step, a KM buffer supplemented with 1% NP-40, 1 mM EGTA, and 5 mM DTT was used. The buffer (0.25 mL) was then added to the dish, incubated for 3 min (min) on ice, and removed. Another 0.5 mL of buffer was added to the dish, incubated for 27 min on ice, and then the extract was combined with the previous extract and marked as the NP-40 fraction. After the first extraction step, the structures on the dish were washed three times with a KM buffer and incubated with 0.25 mL of KM buffer supplemented with DNase I (100 μ/mL; Roche) for 15 min at 37 °C. The extract was removed and marked as the DNase fraction. Structures on the dish were washed three times with a KM buffer and incubated for 30 min on ice in 0.25 mL KM buffer containing 2 M NaCl, 1 mM EGTA, and 5 mM DTT. The extract was removed and marked as the NaCl fraction. After the third extraction step, the structures on the dish were washed three times with a KM buffer and incubated for 30 min at 37 °C in 0.375 mL of KM buffer supplemented with DNase I (100 μ/mL; Roche) and RNase A (5 μ/mL; Serva Electrophoresis GmbH, Heidelberg, Germany). The extract was removed and marked as the DNase/RNase fraction. After the final extraction step, the structures were washed three times with a KM buffer, and the remaining highly insoluble structures were dissolved in a KM buffer containing 1% SDS and marked as the SDS fraction. Proteins in the extracted fractions were precipitated by acetone, dissolved in a Laemmli buffer, and analyzed by SDS electrophoresis.

### 2.7. Immunofluorescence Staining

Cells grown on the coverslips were washed three times in phosphate-buffered saline (PBS; Lonza). Then, the samples were fixed with 3.7% paraformaldehyde in PBS for 15 min and permeabilized in 0.5% Triton X-100 in PBS for 5 min. After being washed in PBS (3 × 10 min), the samples were blocked with 0.25% gelatin and 0.25% bovine serum albumin in PBS for 30 min. Immunostaining with primary and secondary antibodies was carried out for 1 h and 30 min, respectively, with extensive washing in PBS after incubation. Then, the coverslips were briefly washed in deionized water, air dried, and mounted in DAPI Gold solution (Thermo Fisher Scientific). The samples were observed using an Olympus IX71 microscope or confocal microscope Zeiss LSM880.

### 2.8. Western Blot Analysis

The cells were harvested at the indicated times, washed with PBS, and resuspended in an ice-cold RIPA buffer (150 mM NaCl; 5 mM EDTA; 50 mM Tris-HCl pH = 7.4; 0.05% NP-40; 1% sodium deoxycholate; 1% Triton X-100; 0.1% SDS) supplemented with a cocktail of protease inhibitors (Roche). Cell lysis was carried out by incubating the cells for 20 min on ice. Cellular debris was then removed by centrifugation (20,000× *g*, 30 min, 4 °C).

The protein samples were resolved in 10% SDS-PAGE and electro-transferred onto the nitrocellulose membrane in a cooled blotting buffer (0.3% Tris, 1.44% glycine, 20% methanol) at 2.5 mA/cm^2^ for 90 min. The membranes were blocked in 5% non-fat milk in PBS for 1 h. Immunostaining with primary and secondary antibodies was carried out for 1 h and 30 min, respectively, with extensive washing in PBS after incubation. The membranes were developed using an enhanced chemiluminescence reagent (Thermo Fisher Scientific), and the signal was visualized by an Amersham Imager 600 RGB (GE Healthcare, Chicago, IL, USA). When desired, the membrane was re-probed according to [31]. Briefly, the membrane was washed in PBS, incubated in 30% peroxide for 15 min at 37 °C, washed twice in water (15 min each washing), and then incubated for 15 min in PBS, 45 min in 5% non-fat milk in PBS, and stained with antibodies. The band intensities of proteins were assessed using the Amersham Imager 600 RGB software and normalized to GAPDH levels (if not stated otherwise). The fold increase in the protein level was compared with that of the control cells. The analyses of all western blots were performed using non-cropped blots, but some blots presented were cropped for clear presentation purposes.

### 2.9. Microtubule Pelleting Assay

Microtubule pelleting assay was performed according to [12,32]. Infected 3T6 cells (40 hpi) or transfected WOP cells (24 hpt) were lysed in a lysis buffer (20 mM Tris-HCl pH = 6.8; 140 mM NaCl; 0.5% NP-40; 1 mM MgCl_2_; 2 mM EGTA; 4 µg/mL taxol) for 3 min. Lysates were centrifuged for 10 min at 12,000× *g*, 4 °C to obtain the soluble fraction (s) and polymerized fraction (p). The equivalent proportions of the s and p fractions were resolved by SDS-PAGE and immunoblotted with a specific antibody. The band intensity of each protein was determined using the Amersham Imager 600 RGB software, and the percentage of polymerized tubulin was calculated as (p_tubulin_)/(s_tubulin_+p_tubulin_). The fold change of the polymerized tubulin level was compared with the level in the mock-infected or mock-transfected cells, respectively.

### 2.10. VP1/EGFP-tVP3 Pentamer Isolation

The infected cells were harvested at 72 hpi, and VP1/EGFP-tVP3 virus like particles were isolated using the standard protocol [25]. To gain pentamers, the particles were dialyzed at 4 °C for 1.5 h against dissociation buffer I (20 mM Tris-HCl, pH = 8.8; 50 mM NaCl; 2 mM DTT; 5 mM EDTA), followed by 1.5 h dialysis in dissociation buffer II (20 mM Tris-HCl, pH = 8.8; 50 mM NaCl; 5 mM EDTA). Protein aggregates in the sample were pelleted by centrifugation at 20,000× *g*; 4 °C for 30 min.

### 2.11. Direct Immunoelectron Microscopy

The samples (10 µL) were adsorbed on parlodion–carbon-coated grids for 5 min and blocked with 0.5% BSA in PBS for 30 min. Immunostaining with primary and secondary antibodies was carried out for 1 h and 30 min, respectively, with washing in PBS (three times for 3 min) after incubation. Then, the samples were negatively stained for 1 min with 2% phosphotungstic acid two times (pH 7.3 (Sigma-Aldrich)) and air-dried. Electron micrographs were recorded using a JEM-1011 electron microscope (JEOL, Tokio, Japan) operating at 80 kV.

### 2.12. Microtubule Preparations

Tubulin was isolated from pig brains and labeled as described previously [33,34,35]. For the preparation of biotinylated microtubules, isolated tubulin was mixed with biotinylated tubulin (Cytoskeleton Inc.) at a 50:1 mass ratio. For the preparation of rhodamine-labeled microtubules, isolated tubulin was mixed with rhodamine-labeled tubulin (Cytoskeleton Inc.) at a 5:1 mass ratio. GMPCPP-stabilized microtubules were grown using a mixture of 1.3 mg/mL tubulin and 2 mM GMPCPP (guanosine-5′-[(α,β)-methyleno]triphosphate, Merck Millipore, Billerica, MA, USA) in a BRB80 buffer (80 mM Pipes, pH = 6.8, 2 mM MgCl_2_, 1 mM EGTA) and incubated for 1 h for the microtubule binding assay or 15 min for the dynamic microtubule assay at 37 °C. The mixture was then spun at 14,000× *g*. Finally, the supernatant was discarded, and the pellet was resuspended in 50 μL BRB80 buffer.

### 2.13. Total Internal Reflection Fluorescence (TIRF) Microscopy

Imaging was carried out at room temperature using an inverted microscope (Nikon, Ti-E Eclipse) equipped with a 100× or 60× 1.49 N.A. oil immersion objective (Nikon, Plan Apo) and an electron multiplying CCD camera (Andor Technology, iXon Ultra 888). The samples were excited using a LU-N4/N4S laser unit (Nikon). Illumination and image acquisition were controlled by NIS Elements Advanced Research software (Nikon). Rhodamine-labeled microtubules and EGFP-labeled proteins were visualized sequentially by switching between a 561 nm and 488 nm laser and changing the filter set. The acquisition rates were one frame per 10 s. The flow chambers for the TIRF imaging assays were prepared as described previously [36,37]. The channels were treated with an anti-β-tubulin antibody (1 mg/mL in PBS) or anti-biotin (1 mg/mL in PBS) solution for 5 min, followed by one-hour incubation with 1% Pluronic F127 (Sigma-Aldrich). For the microtubule binding assay, the channels were each washed with 40 μL BRB80 buffer with a subsequent injection of rhodamine-labeled microtubules. Then, the channels were flushed with 20 μL BRB80 buffer to remove unbound microtubules. Finally, the flow chamber was suffused with VP1/EGFP-tVP3 pentamers diluted in an assay buffer (BRB80 supplemented with 0.5 mg/mL casein, 10 mM dithiothreitol, 0.1% Tween-20, 20 mM d-glucose, 22.4 μg/mL glucose oxidase, 20 μg/mL catalase, and 1 mM ATP). For the dynamic microtubule assay, the channels were each washed with 40 μL BRB80 buffer with a subsequent injection of biotinylated unlabeled microtubules. Then, the channels were flushed with 20 μL BRB80 buffer to remove unbound microtubules. Finally, the flow chamber was suffused with a mixture of 15 µM rhodamine-labeled tubulin and VP1/EGFP-tVP3 diluted in a polymerization buffer (BRB80 supplemented with 20 mM d-glucose, 22.4 μg/mL glucose oxidase, 20 μg/mL catalase, 1 mM ATP and 1 mM GTP).

### 2.14. Tubulin-VP1 Co-Sedimentation Assay

To perform the VP1 co-sedimentation assay, first 1.8 μg VP1/EGFP-tVP3 and 50 µg unlabeled porcine tubulin were mixed in a 20 μL BRB80 buffer. The mixture was incubated for 10 min at room temperature. The samples were then spun at 50,000× *g*, 30 min. Then, the supernatants were separated from the pellets, and the pellets were resuspended in 20 μL BRB80 buffer. Finally, 10 μL of each supernatant and pellet fraction was analyzed by SDS-PAGE.

### 2.15. Nocodazole Sensitivity Assay

A nocodazole sensitivity assay was performed according to [12]. WOP cells were transfected with pVP1 and treated with 4 µM nocodazole (Merck Millipore) 24 h post transfection for 5, 10, 20, or 60 min. To remove soluble tubulin, cells were rinsed twice with a PHEM buffer (60 mM PIPES; 25 mM HEPES; 4 mM MgSO_4_; 10 mM EGTA; pH = 7.0) and then extracted for 1 min with a PHEM buffer supplemented with 0.2% Triton X-100. The extraction of nocodazole-treated cells was performed in a buffer supplemented with nocodazole at the same concentration used for the treatment to prevent the polymerization of the microtubules during extraction. After the extraction of free tubulin, the cells were fixed, and VP1 and tubulin were stained by specific antibodies.

To quantify the number of microtubules in the cells expressing VP1, the transfected cells were treated with 4 µM nocodazole for 30 min. After the treatment, the cells were scraped into the media and pelleted by centrifugation (5 min; 200× *g*). The cells were resuspended in 250 µL of PHEM buffer supplemented with 0.2% Triton X-100 and a cocktail of protease inhibitors (Roche) and immediately centrifuged for 1 min at 16,000× *g*. A supernatant (containing a soluble tubulin fraction) was designated as the s fraction. The pellet was resuspended in 250 µL ice-cold RIPA buffer, lysed for 20 min on ice, and designated as the p fraction. The equivalent proportions of the s and p fractions were resolved by SDS-PAGE and immunoblot was analyzed with a specific antibody. The band intensity of each protein was determined using the Amersham Imager 600 RGB software, and the percentage of polymerized tubulin was calculated as (p_tubulin_)/(s_tubulin_ + p_tubulin_). The fold change in the level of polymerized tubulin was compared with the level in the mock-treated (*t* = 0 min) cells. Statistical relevance was tested by an independent Student’s two-sample *t*-test with a significance value of *p* ˂ 0.05.

### 2.16. Impact of HDAC6 Inhibition on Viral Protein Levels and Virus Production

Mouse fibroblasts 3T6 and 3T3-wt or αTAT1 KO were infected and24 hpi the media were supplemented with tubacin to achieve a final concentration of 5 µM. The cells were left with tubacin for an additional 16 h or 24 h. Cells were lysed at 40 hpi (16 h treatment). Then, the lysates were resolved in 10% SDS-PAGE gel, transferred to the membrane, and the proteins were detected by specific antibodies. The band intensity of each protein was determined using the Amersham Imager 600 RGB software and normalized to GAPDH levels. The fold increase in the protein level was compared with that in the mock-treated cells. To assess virus production, cells were lysed at 48 hpi (24 h treatment) in a culture media using three freeze–thaw cycles. Cellular debris was removed by centrifugation (8000× *g*; 10 min; 4 °C), and supernatant was used as a virus inoculum in the subsequent experiments. 3T6 cells were infected with equal amount of virus inocula. Then, cells were fixed at 24 hpi, and the LT antigen was stained by specific antibodies. The numbers of infected cells were scored by immunofluorescence microscopy. At least 300 cells were counted for each experiment, and the amounts of infectious viral particles in the viral inocula were assessed. The amount of the virus produced in the tubacin treated cells was compared with that produced in the mock-treated cells.

### 2.17. Significance of Acetylated Microtubules for Viral Proteins and Virus Production

The 3T3-wt, αTAT1 KO, WOP, WOP-EGFP-αTAT1 cells, or WOP cells expressing EGFP, were infected. The cells were lysed at 40 hpi. Then, the lysates were resolved in 10% SDS-PAGE gel, transferred to membrane, and the proteins were detected by specific antibodies. The band intensities of the proteins were determined using the Amersham Imager 600 RGB software and normalized to GAPDH levels. The fold increase in the proteins was compared with their levels in the 3T3-wt or WOP cells. To assess virus production, cells were lysed at 48 hpi in a culture media using three freeze–thaw cycles. Cellular debris was removed by centrifugation (8000× *g*; 10 min; 4 °C), and a supernatant was used as the virus inoculum in the subsequent experiments. 3T6 cells were infected with equal amount of the virus inoculum, cells were fixed at 24 hpi, and the LT antigen was stained with specific antibodies. The numbers of infected cells were scored by immunofluorescence microscopy. At least 300 cells were counted per experiment, and the amount of infectious viral particles in the viral inoculum was assessed. The amount of virus produced in the cells was compared with that produced in the 3T3-wt or WOP cells.

### 2.18. Statistical Analysis

Data are presented as the mean values from three independent experiments, unless otherwise noted. The error bars represent SD. Student’s *t*-test was performed using the GraphPad Prism software, version 6.0 (GraphPad Software, La Jolla, CA, USA). * *p* ˂ 0.05, ** *p* ˂ 0.01, *** *p* ˂ 0.001, **** *p* ˂ 0.0001 were considered to be significant.

## 3. Results

### 3.1. Microtubule Acetylation Gradually Increases during the Late Phase of Infection

**Figure 1:**
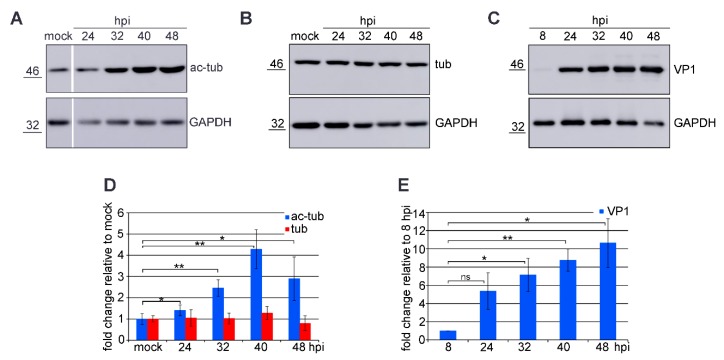
The acetylation of microtubules was elevated during the late phase of infection. (**A**–**C**) 3T6 cells were infected with MPyV and lysed at the indicated hours post infection (hpi). Lysates were separated by SDS/PAGE, transferred onto membrane, and acetylated tubulin (ac-tub), tubulin (tub), VP1, and GAPDH were stained by specific antibodies. (**D**,**E**) A graphic illustration of a densitometry analysis of the digital images of Western blots from four independent experiments. Shown is the fold increase relative to mock-infected cells (a representative 24 h time point) (**D**) or infected cells at 8 hpi (representing amount of incoming virus) (**E**). +/− SD. * *p* < 0.05, ** *p* < 0.01, determined by the Student’s *t* test. The changes in total levels of tubulin (**D**, red columns) were not significant (ns) according to the analysis using the Student’s *t* test.

Our previous research showed that microtubule acetylation is elevated in cells expressing VP1. In this study, we examined microtubule acetylation in the late phase of MPyV infection in detail. Infected cells were harvested in 8-h intervals, and the amounts of tubulin, acetylated α-tubulin, and VP1 were analyzed by Western blot. The levels of acetylated α-tubulin and tubulin were compared with the levels of these proteins in mock infected cells at 24 hpi. The levels of VP1 were related to the VP1 levels at 8 hpi. At this time point, most of the input viral particles became cleared by cells and only particles which were released from endoplasmic reticulum to the cytosol from where their genomes can be delivered to the cell nucleus were present in the cells. Together with increasing levels of VP1, the number of acetylated microtubules increased gradually and reached its peak value at 40 hpi (Figure 1). Although the level of acetylated α-tubulin was elevated, the amount of tubulin remained invariable during the late phase of MPyV infection (Figure 1B,D).

### 3.2. mRNA Levels of HDAC6 and αTAT1 Are Not Affected in the Late Phase of MPyV Infection

**Figure 2:**
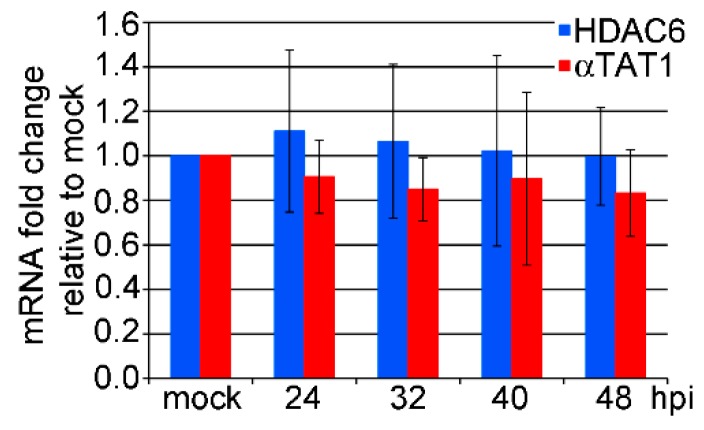
VP1 expression does not change the mRNA levels of either α-tubulin acetyltransferase 1 (αTAT1) or histone deacetylase 6 (HDAC6). 3T6 cells were infected, and the levels of HDAC6 or αTAT1 mRNA were measured by qPCR at the indicated times post infection. Data represent the fold change relative to the mock-infected cells from four independent experiments +/− SD. Data were not significant according to the analysis using Student’s *t* test.

Next, we were interested in whether the elevated level of microtubule acetylation is caused by the deregulation of the expression of deacetylating (HDAC6) and/or acetylating (αTAT1) enzymes. As shown in Figure 2, despite a marked increase in microtubule acetylation, the mRNA levels of both enzymes were not significantly affected during the late phase of infection (Figure 2).

These data show that considerably higher microtubule acetylation is not realized by the deregulation of HDAC6 and/or αTAT1 gene expression but rather by affecting HDAC6 and/or αTAT1 enzymatic activity. Our previous study indicated that VP1-microtubule binding may increase microtubule stability. Furthermore, Coombes et al. [38] observed that microtubule acetylation gradually accumulates on a stable subset of microtubules in the cell. Stable microtubules can then serve as a support for αTAT1 activity, resulting in elevated levels of microtubule acetylation.

### 3.3. Amount of Microtubules is Increased in Cells Expressing VP1

**Figure 3:**
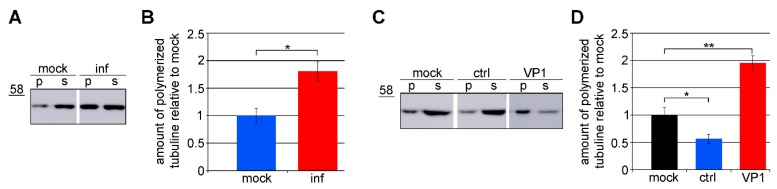
VP1 increases the level of microtubules in cells. (**A**) 3T6 cells were infected or (**C**) WOP cells were transfected with the control plasmid (ctrl) or with the plasmid expressing VP1 (VP1). Cells were fractionated into a polymerized (p) and soluble (s) fraction at 40 hpi or 24 hpt. These fractions were applied to SDS-PAGE, immunoblotted, and tubulin was stained by a specific antibody. (**B**,**D**) A graphic illustration of a densitometry analysis of the digital images of Western blots from three independent experiments. The fold increase in polymerized tubulin relative to the mock-infected/mock-transfected cells is shown. +/− SD. * *p* < 0.05, ** *p* < 0.01 determined by Student’s *t* test.

We next analyzed whether VP1 production elevates the levels of polymerized tubulin in cells, thereby increasing the level of the preferred substrate for αTAT1. MPyV-infected cells and cells expressing VP1 only were used for fractionation. Mouse 3T6 fibroblasts, permissive for MPyV, were used for all experiments in the infection studies. Unfortunately, their transfection efficiency was low (20–25%). Therefore, highly transfectable (85–90%) WOP cells (3T3 fibroblasts constitutively expressing the large T antigen of MPyV) were used for transient VP1 expression studies. After fractionation, the amount of tubulin in the polymerized and soluble fraction was measured by Western blot analysis. In the infected cells, the number of microtubules was approximately two times higher than that in the mock-infected cells (Figure 3A,B). Accordingly, in cells transiently expressing VP1, the number of microtubules was two times higher than that in the mock-transfected cells (Figure 3C,D). These data suggest that VP1 induces the stability of the microtubules affecting their dynamics.

### 3.4. VP1 Binds Microtubules Directly and Stabilizes Them

To investigate whether VP1 binds microtubules directly and how it affects microtubule dynamics, we used an in vitro VP1-microtubule binding assay. As both infectious viral particles [39,40] and VLPs [41] are assembled in the cell’s nucleus after the transportation of capsomeres formed in the cytoplasm, we assumed that in the cytoplasm of cells, VP1 interacts with microtubule structures mostly in the form of capsomeres. For the preparation of capsomeres, VP1/EGFP-tVP3 virus-like particles were isolated from insect cells infected with recombinant baculovirus and disassembled into capsomeres (Figure S1). These particles were designed to contain EGFP as fusion protein with the common short C- terminus sequence of the minor capsid proteins responsible for their interaction with the central cavity of VP1 pentameric capsomeres [25].

**Figure 4:**
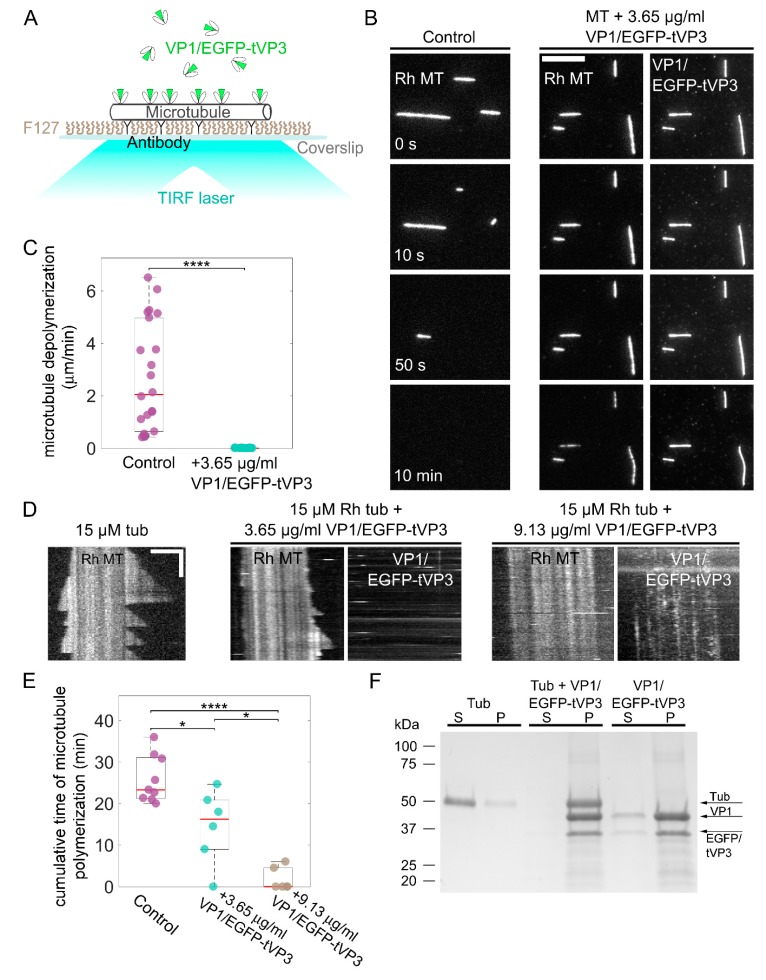
VP1 directly binds microtubules and stabilizes them. (**A**) A schematic diagram of a microtubule binding assay with VP1/EGFP-tVP3. (**B**) Time-lapse fluorescence micrographs of rhodamine-labeled microtubules (Rh MT) depolymerizing in the absence or presence of 3.65 µg/mL VP1/EGFP-tVP3. Scale bar, 10 μm. (**C**) Depolymerization rates of taxol-stabilized rhodamine-labeled microtubules in the absence (control) or presence of 3.65 µg/mL VP1/EGFP-tVP3. Control: 2.67 ± 2.07 µm/min (*N* = 22). Microtubules + 3.65 µg/mL VP1/EGFP-tVP3: 0.01 ± 0.01 µm/min (*N* = 16); values are the mean ± SD. Red lines represent the median values; the bottom and top edges of the box indicate the 25th and 75th percentiles, respectively. The whiskers extend to the extreme data points. (**D**) Kymographs showing dynamic microtubules polymerizing with 15 µM free rhodamine-labeled tubulin in the absence or presence of 3.65 or 9.13 µg/mL VP1/EGFP-tVP3. Horizontal scale bar 3 μm; vertical bar 5 min. (**E**) Cumulative polymerization time of both microtubules’ plus and minus tips over 20 min in the absence or presence of 3.65 or 9.13 µg/mL VP1/EGFP-tVP3. Tubulin (15 µM) control: 25.85 ± 5.68 (*N* = 9), tubulin (15 µM) + 3.65 µg/mL VP1/EGFP-tVP3: 14.5 ± 8.91 (*N* = 6), tubulin (15 µM) + 9.13 µg/mL VP1/EGFP-tVP3: 1.75 ± 2.75 (*N* = 6); values are the mean ± SD. Red lines represent median values; the bottom and top edges of the box indicate the 25th and 75th percentiles, respectively. The whiskers extend to the extreme data points. (**F**) Coomassie-stained SDS-polyacrylamide gel electrophoresis (SDS-PAGE) of 5 mg/mL unlabeled porcine tubulin, 182.5 μg/mL VP1/EGFP-tVP3, or their mixture, after the VP1-co-sedimentation assay. Supernatant fraction (s) and pellet (p). In (**C**) and (**E**), * *p* < 0.05, **** *p* < 0.0001, determined by Student’s *t* test.

To visualize the interaction of VP1/EGFP-tVP3 capsomeres with microtubules, we immobilized the taxol-stabilized rhodamine-labeled microtubules on the surface of the flow chamber with anti-β-tubulin antibodies; then, we flushed in VP1/EGFP-tVP3 diluted in an assay buffer and visualized the system by TIRF microscopy (Figure 4A). At 3.65 µg/mL VP1/EGFP-tVP3, we observed VP1/EGFP-tVP3 binding directly to the microtubules (Figure 4B). No interaction between the isolated EGFP and the microtubules was detected [42]. Next, to test if VP1/EGFP-tVP3 capsomeres stabilize the microtubules, we flushed a taxol-free assay buffer without or with 3.65 µg/mL VP1/EGFP-tVP3 in the channel with taxol-stabilized microtubules. While in the absence of VP1/EGFP-tVP3, the microtubules disassembled rapidly, in the presence of VP1/EGFP-tVP3, the microtubules did not exhibit any observable depolymerization (Figure 4C).

Furthermore, we examined whether VP1 binding affects microtubule dynamics using a microtubule dynamics assay. In the absence or presence of VP1/EGFP-tVP3, we introduced 15 μM rhodamine-labeled porcine tubulin in a polymerization buffer to GMPCPP-stabilized microtubule seeds immobilized on the coverslip and followed this process by TIRF microscopy. In this experiment, we did not observe any VP1/EGFP-tVP3 particles bound to the microtubules at a concentration of 3.65 µg/mL VP1/EGFP-tVP3, although there was a substantial decrease in microtubule polymerization time at both the plus and minus tips (Figure 4D,E). Furthermore, we observed almost no polymerization at a concentration of 9.13 µg/mL VP1/EGFP-tVP3 and only faint binding to the microtubules. This suggests that VP1/EGFP-tVP3 binds free tubulin, depleting it from the solution and hindering microtubule dynamics. To prove this, we performed a co-sedimentation assay. We incubated a mixture of VP1/EGFP-tVP3 with unlabeled porcine tubulin, sedimented it by centrifugation, and analyzed the sediment and supernatant by SDS-PAGE. While the tubulin without the presence of VP1/EGFP-tVP3 remained mostly in the supernatant, both components of the mixture of VP1/EGFP-tVP3 with tubulin appeared fully in the pellet (Figure 4F).

**Figure 5:**
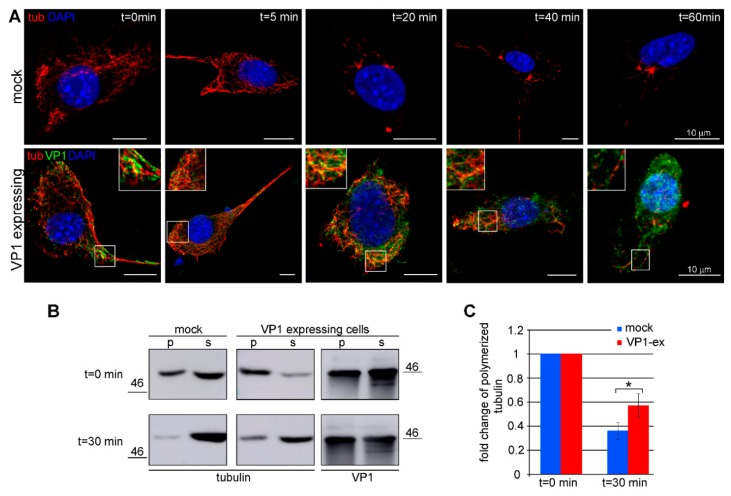
VP1 protects microtubules from nocodazole induced depolymerization. WOP cells were transfected with plasmids expressing VP1 and 24 hpt cells were treated with nocodazole (4 µM) for the indicated times. (**A**) After the treatment, soluble tubulin was washed out, the cells were fixed, and polymerized tubulin (tub; red) and VP1 (green) were stained by specific antibodies. Shown are the selected confocal sections. Enlarged details of the cells are presented in insets. Bar: 10 µm. (**B**) After the treatment, cells were fractionated into a soluble tubulin fraction (s) and polymerized tubulin fraction (p). Equal amounts of the fractions were resolved via SDS/PAGE, immunoblotted, and tubulin and VP1 were stained with specific antibodies. (**C**) A graphic illustration of a densitometry analysis of the digital images of Western blots from three independent experiments. The fold change in polymerized tubulin relative to the mock-treated cells (*t* = 0 min) is shown. +/− SD; * *p* ˂ 0.05 determined by Student’s *t* test.

The stability of microtubules was further confirmed at a cellular level using a nocodazole sensitivity assay. VP1-expressing cells were treated with the microtubule-destabilizing drug nocodazole for 5, 20, 40, and 60 min. After the treatment, cells were extracted to remove free tubulin, whose bright diffuse staining would otherwise obscure the microtubules [12]. The extracted cells were fixed, and the microtubules and VP1 were stained by specific antibodies. The microtubules were observed in non-treated cells (*t* = 0 min) and in cells treated for 5 min with nocodazole in cells expressing VP1, as well as in the control mock-transfected cells (Figure 5A). During the course of exposure to nocodazole, almost all microtubules disappeared in the mock-transfected cells, while many microtubules were observed in VP1-expressing cells (Figure 5A). To quantify microtubule depletion, the total number of microtubules remaining in the cells after 30 min of nocodazole treatment was measured by Western blot. In the mock-transfected cells, the amount of microtubules declined by 64%, whereas, in VP1-expressing cells, the amount of microtubules declined by only 43% (Figure 5B,C).

Taken together, these data show that VP1 binds microtubules (and tubulin) directly, which leads to their stabilization and an increase in the microtubule level within the cells.

### 3.5. VP1 Stimulates Hyperacetylation of Microtubules

**Figure 6:**
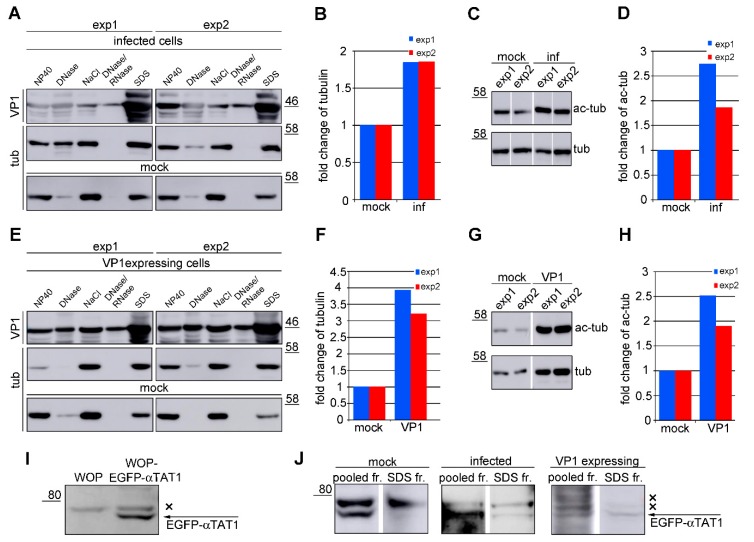
αTAT1 is a part of the VP1–hyperacetylated microtubule complex. (**A**,**E**) Infected 3T6 cells (**A**) or VP1 expressing cells (**E**) were in situ fractionated; then, an equal amount of washed out material from each fraction was separated via SDS/PAGE and transferred onto the membrane. The presence of VP1 and tubulin (tub) in each fraction was determined by specific antibodies. (**B**,**F**) A graphic illustration of the densitometry analysis of the digital images of Western blots from two independent experiments. Presented are the fold changes of tubulin in the SDS fraction of the infected (**B**) or VP1-expressing cells (**F**), which were compared with mock-infected or mock-transfected cells. (**C**,**G**) An equal amount of the SDS fraction from infected (inf) (**C**) or VP1-expressing cells (VP1) (**G**) was resolved on SDS/PAGE, immunoblotted to the membrane and tubulin, and acetylated α-tubulin (ac-tub) was stained by specific antibodies. (**D**,**H**) A graphic illustration of the densitometry analysis of the digital images of Western blots from two independent experiments. Presented are the fold changes of α-tubulin acetylation ratios in the SDS fraction of the infected (**D**) or VP1-expressing cells (**H**), which were compared with those of the mock-infected or mock-transfected cells. (**I**) Lysates of WOP and WOP-EGFP-αTAT1 were separated by SDS/PAGE, blotted onto the membrane, and EGFP was stained by a specific antibody. (**J**) Infected (inf) or VP1-expessing cells were in situ fractionated, and equal amounts of the pooled fractions and SDS fraction were separated by SDS/PAGE, transferred onto the membrane, and EGFP was stained by a specific antibody; × indicates non-specific antibody staining.

In a previous study [3], we performed an in situ fractionation of infected cells and showed that a subpopulation of tubulin accumulates in the last insoluble fraction together with VP1. This method is based on the successive washing out of proteins according to their solubility in different buffers. The last fraction, soluble only by SDS, contains highly insoluble protein complexes. Here, we address whether the tubulin in the VP1–microtubule insoluble complex is more acetylated than the last fraction of microtubules in the control (VP1 negative) cells. Therefore, the VP1 positive and control cells were again in situ fractionated, and each fraction was analyzed by Western blot to determine the levels of VP1 and tubulin. VP1 was detected in all fractions of both infected cells and cells transiently producing VP1. The highest VP1 level was detected in the last SDS fraction (Figure 6A,E). Although tubulin was detected in the SDS fraction in the mock-infected or mock-transfected cells, the amount of tubulin was significantly higher in the VP1-producing cells (by almost two times in the infected and four times in the transfected cells) (Figure 6A,B,E,F). Western blot analysis of the SDS solubilized fractions with the antibody directed against acetylated α-tubulin revealed that the level of acetylated tubulin was 2.5-times higher in the VP1 positive cells compared to that in the control (VP1 negative) cells (Figure 6C,D,G,H).

**Figure 7:**
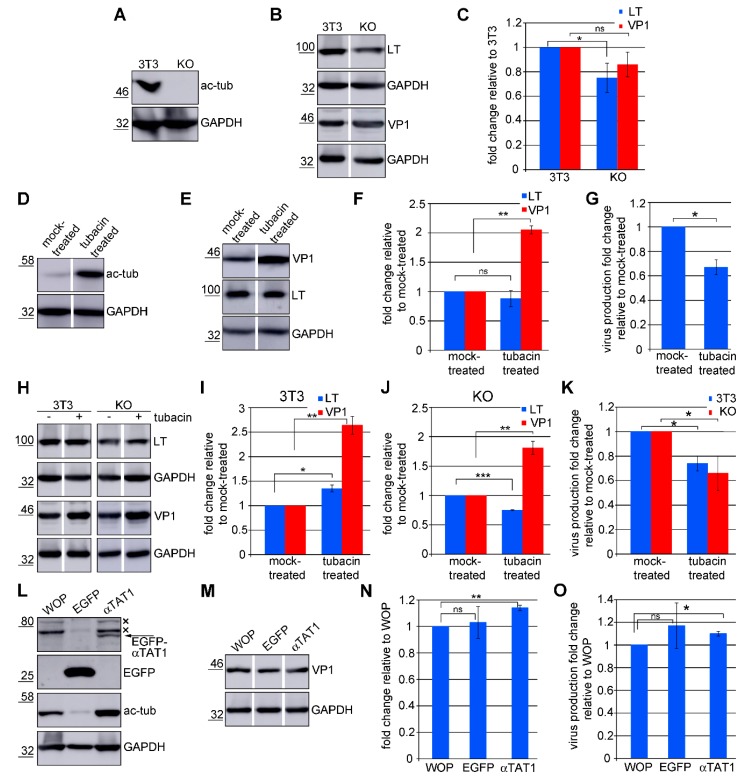
Acetylated microtubules are dispensable for MPyV infection. (**A**) Lysates of 3T3-wt and αTAT1 KO cells were separated by SDS/PAGE, blotted onto the membrane, and acetylated α-tubulin (ac-tub) and GAPDH were stained by specific antibodies. (**B**) Lysates (40 hpi) of infected 3T3-wt and αTAT1 KO cells were resolved on SDS/PAGE, transferred onto the membrane, and VP1, LT, and GAPDH were stained by specific antibodies. (**C**) A graphic illustration of the densitometry analysis of the digital images of Western blots of three independent experiments. The fold change relative to 3T3-wt cells +/− SD is shown. (**D**) Lysates of 3T6 cells treated for 24 h with 5 µM tubacin were separated by 10% SDS/PAGE, transferred onto a membrane, and acetylated α-tubulin and GAPDH were stained by specific antibodies. (**E**,**H**) 3T6 (**E**), 3T3-wt, and αTAT1 KO cells (**H**) were infected, and 24 hpi tubacin was added (5 µM). Cells were lysed after 16 h tubacin treatment, lysates were separated by SDS/PAGE and immunoblotted, and VP1, LT, and GAPDH were stained by specific antibodies. (**F**,**I**,**J**) Graphic illustration of the densitometry analysis of the digital images of the Western blots of three independent experiments. Shown is the fold change relative to the mock treated 3T6 (**F**), 3T3-wt, (**I**) and αTAT1 KO (**J**) cells +/− SD. (**G**,**K**) Cells 3T6 (**G**), 3T3-wt, and αTAT1 KO cells (**K**) were infected; then, 24 hpi tubacin was added (5 µM), and, after 24 h of incubation, MPyV virions were isolated. 3T6 cells were infected with equal volumes of the virus isolated from the tubacin treated or mock-treated cells. Cells were fixed at 24 hpi, and the LT antigen was stained by a specific antibody. The values in the graph refer to the fold change in numbers of LT positive cells relative to the mock-treated cells and represent the mean values from three independent experiments +/− SD. (**L**) Lysates of WOP, WOP-EGFP-αTAT1 (αTAT1), and WOP-EGFP expressing cells (EGFP) were separated by SDS/PAGE, blotted onto the membrane, and acetylated α-tubulin, EGFP, and GAPDH were stained by specific antibodies. (**M**) Lysates of infected WOP, WOP-EGFP-αTAT1, and WOP-EGFP expressing cells were performed at 40 hpi and resolved by SDS/PAGE; proteins transferred onto the membrane and VP1 and GAPDH were stained by specific antibodies. (**N**) Graphic illustration of the densitometry analysis of the digital images of Western blots of three independent experiments. Shown is the fold change relative to the WOP cells +/− SD. (**O**) WOP, WOP-EGFP-αTAT1, and WOP-EGFP expressing cells were infected, and 48 hpi MPyV virions were isolated. 3T6 cells were infected with equal volumes of the isolated virus from cells. Cells were fixed at 24 hpi, and the LT antigen was stained by a specific antibody. The values in the graph refer to the fold change in the numbers of LT antigen positive cells relative to WOP cells and represent the mean values of three independent experiments +/− SD × indicate non-specific antibody staining. * *p* ˂ 0.05, ** *p* ˂ 0.01, *** *p* ˂ 0.001 determined by the Student’s *t* test, ns—not significant.

We were further interested in whether αTAT1 is a part of the VP1-microtubule complex. To easily track αTAT1 in cells, a WOP cell line stably overexpressing αTAT1 fused with EGFP was established. The overexpression of αTAT1 in WOP-EGFP-αTAT1 cells was confirmed by a Western blot analysis of cell lysates using an antibody specific to EGFP (Figure 6I, Figure 7L). WOP-EGFP-αTAT1 cells were in situ fractionated, and the presence of αTAT1 was examined. EGFP-αTAT1 was detected convincingly in only the last insoluble fraction. As seen in Figure 6J, EGFP-αTAT1 was detected in the SDS fractions of VP1 positive cells but not in mock infected or transfected cells. Nevertheless, in pooled fractions, EGFP-αTAT1 could be detected in both VP1-negative mock-infected cells and VP1-producing infected or transfected cells (Figure 6J). These data show that microtubules in the VP1-microtubule complex are acetylated. This high acetylation is obviously supported by αTAT1, which is also part of the complex, and its activity is stimulated by VP1-mediated microtubule stabilization.

### 3.6. Acetylated Microtubules Are Not Necessary for Viral Infection

Finally, we addressed the role of microtubule acetylation in MPyV infection. To determine whether the absence of microtubule acetylation affects MPyV infection, the levels of LT and VP1 proteins and virus production were tested in αTAT1 KO cells. These cells do not express the major tubulin acetyltransferase, tubulin acetyltransferase 1, and lack acetylated microtubules [23]. The absence of acetylated α-tubulin in αTAT1 KO cells was confirmed by a Western blot analysis of cell lysates using the antibody specific to acetylated α-tubulin (Figure 7A). The levels of LT antigen (relative to GAPDH levels) and VP1 at 40 hpi were only slightly decreased in αTAT1 KO cells compared to their wild-type counterparts (Figure 7B,C). We failed to compare the amounts of virus progeny at 48 hpi in 3T3-wt and αTAT1 KO cells. The problem we encountered was the increased proliferation of αTAT1 KO cells in comparison with the parental cell line. In addition, the altered phosphorylation capacity of αTAT1 KO cells due to deregulation of the Hippo pathway [23] may substantially affect virion production, as virion assembly is positively affected by VP1 phosphorylation [43]. This fact may distort the interpretation of the results.

Furthermore, we artificially elevated the amount of acetylated tubulin by tubacin, a specific inhibitor of HDAC6 [44], and examined the levels of LT antigen, VP1, and viral production at 48 hpi. The increase in tubulin acetylation in cells after tubacin treatment was confirmed by a Western blot analysis of cell lysates using an antibody specific to acetylated α-tubulin (Figure 7D). The amount of the LT antigen was not significantly affected by tubacin treatment (Figure 7E,F), indicating that elevated levels of tubulin acetylation do not restrict early antigen production. However, the level of VP1 in tubacin treated cells was two times higher compared with that of non-treated cells. Surprisingly, despite the increased level of VP1 in tubacin treated cells, the amount of virion progeny dropped to 67% in comparison with the virions produced by the mock-treated cells (Figure 7E–G).

Histone deacetylase 6 is a protein with a wide range of actions. In addition to deacetylase activity, it also possesses a ubiquitin binding domain at its C-terminus and thus affects protein metabolism [45]. Moreover, HDAC6 also deacetylates other proteins besides α-tubulin, such as chaperones, and influences their functions [46,47]. Therefore, the observed inhibition effect of HDAC6 on VP1 levels and virion production is not necessarily connected with elevated levels of microtubule acetylation but may reflect the inhibition or activation of other HDAC6 functions. To determine this, we tested the impact of HDAC6 inhibition on the amount of the LT antigen, VP1, and virus production in infected αTAT1 KO cells. Since αTAT1 KO cells lack αTAT1, the inhibition of HDAC6 cannot affect microtubule acetylation. After tubacin treatment, the αTAT1 KO cells and their wt-counterparts exhibited similar effects to the 3T6 cells in the previous experiment. The amount of LT was not significantly affected by HDAC6 inhibition, whereas the level of VP1 was almost 2.5 or 2 times higher compared to the mock-treated 3T3 or αTAT1 KO cells, respectively (Figure 7H–J). Also, the amount of infectious virus produced by tubacin-treated 3T3 and αTAT1 KO cells dropped to 74% and 66%, respectively (Figure 7K). These data show that the observed impact of HDAC6 inhibition was not a consequence of the elevation of microtubule acetylation but a result of the inhibition of other HDAC6 functions.

To further test the relevance of microtubule acetylation in MPyV infection, the amount of LT antigen, VP1, and viral production was examined in WOP-EGFP-αTAT1 cells overexpressing EGFP-αTAT1. These cells, due to the overexpression of the major tubulin acetyltransferase, exhibited elevated levels of tubulin acetylation. The overexpression of αTAT1 and elevated level of acetylated α-tubulin in WOP-EGFP-αTAT1 cells was confirmed by a Western blot analysis of cell lysates using an antibody specific to EGFP or acetylated α-tubulin (Figure 6I, Figure 7L). We showed that increased levels of microtubule acetylation influenced neither VP1 levels nor the production of progeny virions (Figure 7M–O).

Taken together, these data indicate that microtubule hyperacetylation is not essential for MPyV infection. Additionally, the data show that the inhibition of HDAC6 has a significant impact on MPyV infection, but the reasons behind this effect require further examination.

## 4. Discussion

Microtubules are crucial structures within cells. They are absolutely essential for the intracellular transport of organelles, secretory vesicles, and macromolecular complexes. In addition, microtubules are involved in cell division and cell movement; also, they maintain cell structure and cell polarity. In order to perform these diverse functions, cells build microtubule meshes with various morphologies and dynamics. This diversity is generated through the expression of different tubulin isoforms and posttranslational modifications.

The role of the microtubular network in virus infection has been intensively studied and, in the case of many viruses, well characterized [48]. Furthermore, the relevance of the microtubular network in polyomavirus virion entry and trafficking inside the host cells has been well described [49,50,51]. However, the role of the microtubular network in the late stages of polyomavirus infection and the significance of microtubule posttranslational modifications to infection remain poorly understood.

In this study, we focused on characterizing the relationship of VP1–microtubule interactions, their hyperacetylation and stability, and the functional meaning of elevated microtubule αK40 acetylation during the late phase of MPyV infection. We showed that tubulin αK40 acetylation increased gradually in the late phase of infection depending on the amount of VP1 in infected cells. Also, in transfected cells expressing VP1, the level of αK40 tubulin acetylation increased with increasing level of VP1 production [3]. Consequently, VP1 may be involved in the deregulation of microtubule αK40 acetylation. Although the changes in αK40 tubulin acetylation during viral infection have been described for many viruses [52] how viral infection affects the αK40 tubulin acetylation level is still poorly characterized. One way to alter microtubule acetylation is to affect HDAC6. The envelope protein gp120 of human immunodeficiency virus 1 (HIV-1) increased the level of HDAC6 in primary rat neurons [53], while influenza A virus reduced the level of HDAC6 in infected cells [54]. We showed that MPyV infection does not affect the mRNA levels of either HDAC6 or αTAT1. Therefore, we further tested possible stimulation of αTAT1 activity.

According to the model of Coombes et al. [38], αTAT1 stochastically enters the lumen at microtubule ends or through breaks in the lattice. Then, the mobility of αTAT1 within the lumen is controlled by the affinity of αTAT1 to its binding sites, which are highly concentrated within the lumen [38]. Several conditions may enhance αTAT1 activity: (i) the number of microtubules ends, (ii) the numbers of breaks and openings in the lattice, and (iii) microtubule stability. Despite the slow rate of α-tubulin acetylation in the lumens of microtubules, αTAT1 may be able to slowly but efficiently acetylate stable microtubules while traveling down the lumen [38]. In VP1-positive cells, an increased level of microtubules was observed, and the VP1-mediated stabilization of microtubules was demonstrated, both in vitro and in vivo. The increased number of stable microtubules in cells may provide a substrate for αTAT1 acetylation along the entire length of the microtubules. The proposed model may be enhanced by other processes. We cannot, for example, rule out that VP1 stabilization may lead to increased breakage in the microtubule lattice, thus increasing αTAT1 activity. It was reported that the stabilization of microtubules by taxol led to more open, sheet like microtubule structures [38]. On the contrary, the HIV-1 envelope protein gp120 was shown to interact with tubulin β-3 to prevent microtubule polymerization, which led to a decrease in microtubule αK40 acetylation [55]. We showed that VP1 interferes with microtubule polymerization in vitro, but the significance of this phenomenon in living cells, as well as its influence on microtubule stabilization, needs to be elucidated.

Alterations of microtubule dynamics, accompanied by αK40 acetylation changes, have been described as necessary for several viruses. The stabilization of microtubules is involved in virus trafficking in the cell. Entering HIV-1 particles induce microtubule stabilization for efficient translocation through cytoplasm [56]. The infection of adenoviruses or mouse norovirus 1 leads to stable microtubule reorganization [57,58]. The VP1 of the mouse polyomavirus binds the microtubules, including the mitotic spindle, which results in blocking the cell cycle in the G2/M phase [3]. The reorganization of microtubules is related to rearrangements of other parts of the cytoskeleton, leading to changes in the mechanical properties of the cells. It has been shown that early T antigens of polyomaviruses affect the different components of cytoskeleton networks. The constitutive expression of the LT antigen of the Simian virus 40 resulted in a collapse of the vimentin network followed by the down-regulation of microtubule αK40 acetylation and the re-localization of acetylated microtubules to the center of the cells. These cytoskeletal rearrangements led to an increase in cell stiffness at the cell periphery [59]. Likewise, the small T antigen of the Merkel cell polyomavirus destabilizes microtubules and increases cell mobility [60]. In the late phase of MPyV infection, VP1-induced microtubule stabilization followed by increased acetylation may compensate for the destabilization effects of early T antigens to preserve cell shape. Also, the observed blocking of the cell cycle in the G2/M phase and the prolonged S-phase are apparently important factors for completion of the MPyV infection cycle.

The stabilization of microtubules and their acetylation favors motor binding, thus enhancing their movement along the microtubules [53,61]. The disruption of microtubules by nocodazole inhibited the release of MPyV from infected cells [50], suggesting that intact and likely stable microtubules are indispensable for the active release of MPyV progeny from infected cells. Although our experiments indicated that the αK40 acetylation of microtubules per se is not required for the late phase of MPyV infection, the positive effects of microtubule stabilization and acetylation on the movement of viral components cannot be excluded.

The role of the HDAC6 enzyme in MPyV infection might be more complex. Although we did not observe HDAC6 involvement in microtubule acetylation, our results indicate that HDAC6 is involved in the late phase of MPyV infection. The inhibition of HDAC6 by tubacin resulted in increased levels of VP1 and a decrease in the production of viral progeny. Besides α-tubulin, HDAC6 also interacts with other substrates, including chaperones [47]. Moreover, apart from its deacetylation activity, HDAC6 binds ubiquitinylated proteins [45,62] and thus plays a crucial role in protein metabolism [63].

HDAC6 may regulate the MPyV lifecycle indirectly via its substrates, especially chaperones. HDAC6 is responsible for Hsp90 activity, as the hyperacetylation of Hsp90 results in the unsatisfactory binding of client proteins [46,64]. It was demonstrated that the polymerase of the Japanese encephalitis virus needs to be stabilized by Hsp90. The inhibition of HDAC6 leads to Hsp90 hyperacetylation, polymerase destabilization, and decreases viral replication [65]. Chaperones are involved in the MPyV life cycle, so their activity maintenance by HDAC6 may be important for viral propagation. Moreover, the VP1 protein is acetylated [66] and also ubiquitinylated (our unpublished data). Thus, VP1 can serve as a substrate for HDAC6. HDAC6 may participate in the late phase of infection in various ways. It may directly regulate the metabolism of MPyV structural proteins and the viral assembly. Indirectly, it can affect chaperone activation. Further research is required to characterize the function of HDAC6 in the polyomavirus life cycle and reveal whether HDAC6 is a promising target for the antiviral therapy of diseases associated with polyomaviruses.

## Supplementary Materials

The following are available online at https://www.mdpi.com/1999-4915/12/2/227/s1, Figure S1: Characterization of VP1/EGFP-tVP3 virus-like particles and capsomeres.
