# Supplementary material for: The Major Capsid Protein, VP1, of the Mouse Polyomavirus Stimulates the Activity of Tubulin Acetyltransferase 1 by Microtubule Stabilization
Source: Viruses. 2020 Feb 18;12(2):227. doi: 10.3390/v12020227 (PMC7077302; doi:10.3390/v12020227)
Supplement: Supplementary file 1 [file viruses-12-00227-s001.pdf]

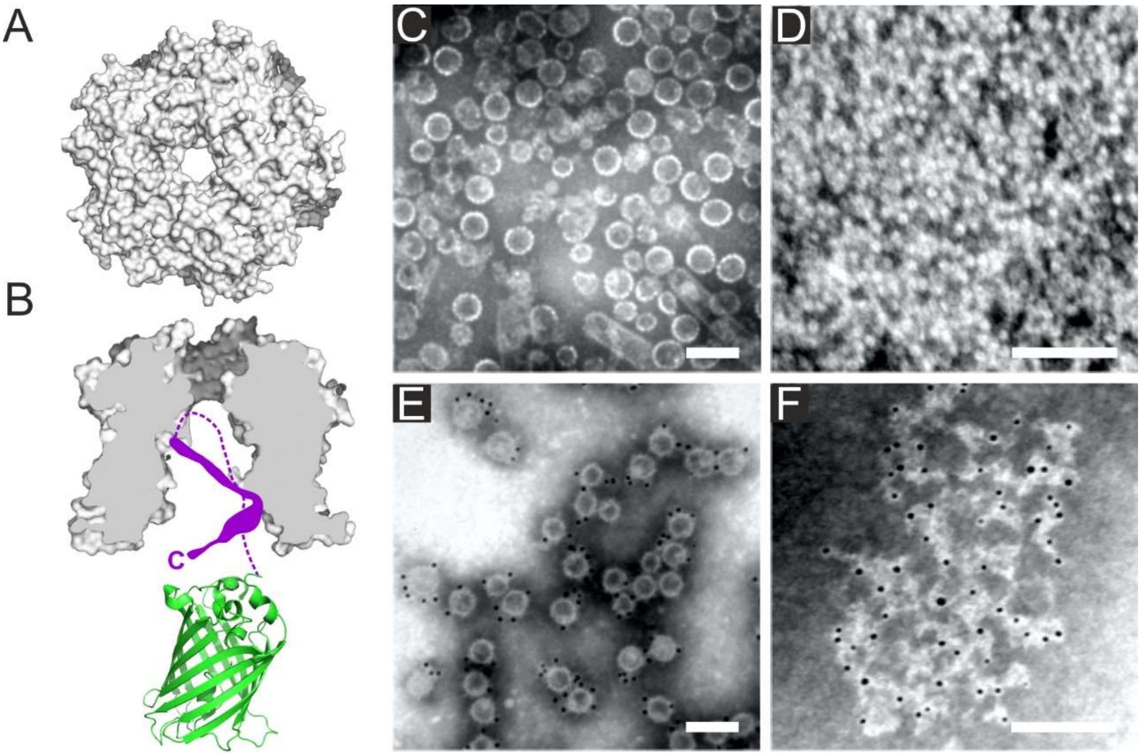

Figure S1. Characterization of VP1/EGFP-tVP3 virus-like particles and capsomeres. (A) Schema of VP1 pentamer, top view. (B) Schematic longitudinal section of the VP1/EGFP-tVP3 capsomere, VP1 protein – grey, truncated VP3 – purple and EGFP – green. (C, D) Negative staining of VP1/tVP3-EGFP particles (C) and capsomeres (D). (E) Direct immunoelectron microscopy of VP1/tVP3-EGFP particles. VP1 protein was stained with specific primary antibody followed by secondary antibody conjugated with 5 nm gold particles. (F) Direct immunoelectron microscopy of VP1/tVP3-EGFP capsomeres. EGFP protein was stained with specific primary antibody followed by secondary antibody conjugated with 5 nm gold particles. Bar 100 nm
